# Ancient Introgression between Two Ape Malaria Parasite Species

**DOI:** 10.1093/gbe/evz244

**Published:** 2019-11-07

**Authors:** Lindsey J Plenderleith, Weimin Liu, Gerald H Learn, Dorothy E Loy, Sheri Speede, Crickette M Sanz, David B Morgan, Paco Bertolani, John A Hart, Terese B Hart, Beatrice H Hahn, Paul M Sharp

**Affiliations:** 1 Institute of Evolutionary Biology, and Centre for Immunity, Infection and Evolution, University of Edinburgh, Edinburgh, United Kingdom; 2 Department of Medicine, University of Pennsylvania; 3 Department of Microbiology, University of Pennsylvania; 4 Sanaga-Yong Chimpanzee Rescue Center, International Development Association-Africa, Portland, Oregon; 5 Department of Anthropology, Washington University in St. Louis; 6 Wildlife Conservation Society, Congo Program, Brazzaville, Republic of the Congo; 7 Lester E. Fisher Center for the Study and Conservation of Apes, Lincoln Park Zoo, Chicago, Illinois; 8 Leverhulme Centre for Human Evolutionary Studies, University of Cambridge, Cambridge, United Kingdom; 9 Lukuru Wildlife Research Foundation, Tshuapa-Lomami-Lualaba Project, Kinshasa, Democratic Republic of the Congo

**Keywords:** chimpanzee, *Plasmodium*, *Laverania*, exported proteins

## Abstract

The *Laverania* clade comprises the human malaria parasite *Plasmodium falciparum* as well as at least seven additional parasite species that infect wild African apes. A recent analysis of *Laverania* genome sequences (Otto TD, et al. 2018. Genomes of all known members of a *Plasmodium* subgenus reveal paths to virulent human malaria. Nat Microbiol. 3: 687–697) reported three instances of interspecies gene transfer, one of which had previously been described. Generating gene sequences from additional ape parasites and re-examining sequencing reads generated in the Otto et al. study, we identified one of the newly described gene transfers as an assembly artifact of sequences derived from a sample coinfected by two parasite species. The second gene transfer between ancestors of two divergent chimpanzee parasite lineages was confirmed, but involved a much larger number of genes than originally described, many of which encode exported proteins that remodel, or bind to, erythrocytes. Because successful hybridization between *Laverania* species is very rare, it will be important to determine to what extent these gene transfers have shaped their host interactions.

The human malaria parasite *Plasmodium falciparum* emerged from the *Laverania* clade, a group of ape *Plasmodium* parasites comprising at least seven cryptic species infecting chimpanzees, gorillas and bonobos (Ollomo et al. 2009; Liu et al. 2010, 2017). *Laverania* are generally host specific (Liu et al. 2010, 2017; Prugnolle et al. 2011; Loy et al. 2017) (fig. 1*a* and supplementary note 1, Supplementary Material online), but hosts are frequently coinfected with multiple parasite species (Liu et al. 2010, 2017; Otto et al. 2018) and *Anopheles* vectors are promiscuous in transmission (Makanga et al. 2016). To understand the factors that define *Laverania* species and host tropism, Otto et al. (2018) recently determined genome sequences from six *Laverania* species and reported that “interspecific gene transfers… were important in the evolution of these species.” This conclusion was based on three examples of anomalous phylogenetic relationships, one previously described (Sundararaman et al. 2016) and two newly reported. Because gene transfers have the potential to shape parasite–host interactions (Sundararaman et al. 2016), we followed up on these findings, generating sequences from additional ape parasites, including the *Laverania* species that infects wild bonobos (Liu et al. 2017). We found that one of the putative new gene transfers likely arose from assembly artifacts. The other was validated, but found to involve not just 3 genes, but as many as 24, across multiple chromosomes. These findings provide evidence for introgression between the ancestors of two divergent species of chimpanzee parasites.

**Fig. 1.**
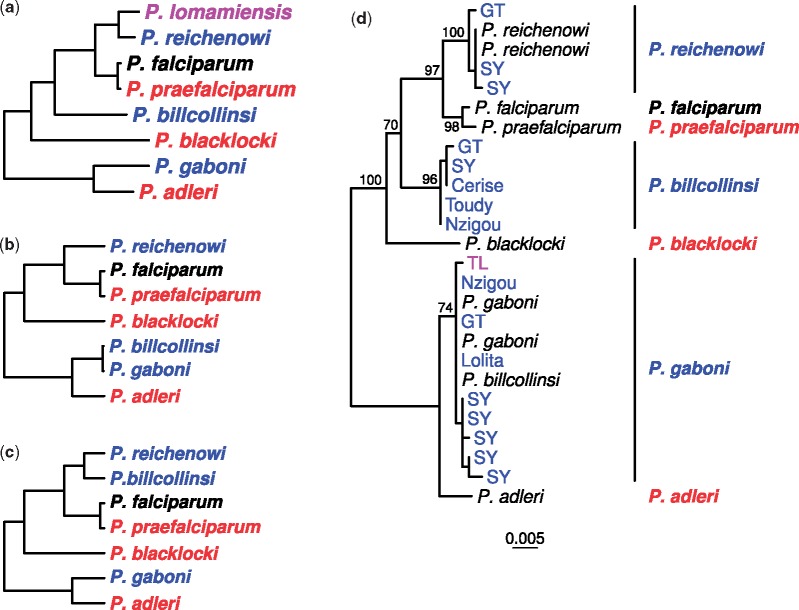
—Relationships among *Laverania* species. (*a*) The standard topology for the eight known *Laverania* species (Liu et al. 2010, 2016, 2017). Colors indicate the parasite’s usual host species: Bonobos (purple), chimpanzees (blue), humans (black), and gorillas (red). Although most prevalent in chimpanzees, *P. gaboni* (and on rare occasions *P. reichenowi*) also infects bonobos (Liu et al. 2017). The tree was derived by maximum likelihood analysis of a 3.4 kb region of the mitochondrial genome (see Supplementary Fig. 1*a* in Liu et al. 2010). (*b*) Schematic representation of an anomalous topology with transfer from *P. gaboni* to *P. billcollinsi* (termed “topology C” in Supplementary Figure 5 of Otto et al. 2018). (*c*) Schematic representation of an anomalous topology with transfer between *P. reichenowi* and *P. billcollinsi* (termed “topology D” in Supplementary Figure 5 of Otto et al. 2018). (*d*) Maximum likelihood tree of one of four genes (gene ID PF3D7_1328000) hypothesized to have resulted from *P. gaboni* to *P. billcollinsi* gene transfer (Otto et al. 2018). Sequences were obtained from published reference genomes (black), limiting dilution PCR from ape blood or fecal samples collected at three field sites (SY, GT, TL), or de novo assembled contigs of sequencing data released by Otto et al. (labelled with the name of the animal from which the data were derived: Toudy, Nzigou, Cerise, Lolita; see Supplementary Table 1 of Otto et al. 2018). For PCR-derived sequences and new contigs, color indicates whether the sample was obtained from a chimpanzee (blue) or a bonobo (purple). Note that the sequence from the published *P. billcollinsi* reference genome clusters within *P. gaboni*, whereas other *P. billcollinsi* sequences fall in the expected position within the tree. The scale bar represents 0.005 nucleotide substitutions per site; bootstrap values from 100 replicates are shown for interspecific branches with at least 70% support.

Gene transfer between species can be inferred when a gene falls in an unusual position within the phylogenetic tree. Previous genome comparisons (Sundararaman et al. 2016) revealed that four adjacent genes on chromosome 4 had likely been transferred from the ancestor of one gorilla parasite (*Plasmodiumadleri*) to that of another (*Plasmodiumpraefalciparum*, the source of *Plasmodiumfalciparum*); because this region includes genes encoding essential, host-specific invasion ligands (Galaway et al. 2017), this may have predisposed the ancestral parasite to cross-species transmission from gorillas to humans (Liu et al. 2010; Wright and Rayner 2014; Otto et al. 2018; Plenderleith et al. 2018). Otto et al. (2018) described two additional topologies that did not follow the species tree (fig. 1*a*): one suggesting transfer of four genes from *Plasmodiumgaboni* into *Plasmodiumbillcollinsi* (fig. 1*b*, referred to as “topology C” in Supplementary Table 5 of Otto et al. 2018) and the other indicating transfer of three genes between *Plasmodiumreichenowi* and *P. billcollinsi* (fig. 1*c*, “topology D” in Otto et al. 2018).

For the four genes with transfer suggested from *P. gaboni* to *P. billcollinsi* (fig. 1*b*), sequences from the published genomes for both species were near-identical, implying very recent divergence. To examine this further, we amplified these loci from additional ape parasites, focusing on samples previously shown to contain *P. billcollinsi*. One of the four genes could not be amplified, but several sequences were recovered for each of the others. In phylogenetic analyses, these sequences formed a lineage at the position expected for *P. billcollinsi* (fig. 1*d* and supplementary fig. 1, Supplementary Material online), rather than clustering with *P. gaboni*.

Because the *P. billcollinsi* reference genome generated by Otto *et al.* was derived from a chimpanzee also infected with *P. gaboni*, we reasoned that the incongruent topology may have arisen from inadvertent misincorporation of *P. gaboni* sequences into the *P. billcollinsi* assembly. We therefore extracted sequencing reads mapping to the anomalous genes in four chimpanzee data sets from Otto et al. (2018) containing *P. billcollinsi*-derived sequences, and performed de novo assembly. For every gene, in addition to *P. gaboni* and *P. reichenowi* sequences, at least one data set yielded a contig that fell within the expected *P. billcollinsi* clade amplified directly from chimpanzee samples (fig. 1). Thus, these contigs and the newly generated chimpanzee-derived amplicon sequences presumably represent the true *P. billcollinsi* sequence, whereas the four incongruent genes in the *P. billcollinsi* reference genome most likely represent assembly artifacts.

To determine whether sequences displaying the anomalous *P. billcollinsi–P. reichenowi* topology (fig. 1*c*) also represented assembly artifacts, we amplified the three genes of interest from additional ape samples; these new sequences also showed *P. billcollinsi* and *P. reichenowi* clustering together (supplementary figs. 2*c*, 2*d*, and 3*h*, Supplementary Material online), rather than following the standard species topology (fig. 1*a*). We noted that genes adjacent to these three loci were not on the list of orthologues screened for anomalous topologies by Otto et al. (2018). They used very stringent criteria in identifying potential interspecies gene transfers; for example, in restricting their analyses to one-to-one orthologues they excluded many members of multigene families. However, we were able to identify additional orthologues based on their location in chromosome alignments, and generated trees for these genes from published and newly-amplified sequences. This approach revealed that the two genes originally identified on chromosome 1 with the anomalous topology were part of a four-gene cluster (*eba-181* region, fig. 2*a*), and five additional genes near *fikk9.7* on chromosome 9 also exhibited the same anomalous topology, although in two clusters, separated by two genes with the standard topology (fig. 2*b*). On both chromosomes, the clusters were delineated at one end by the breakdown of synteny between species found in subtelomeric regions, making it impossible to identify further orthologues. Elsewhere we were able to map the extent of the anomalous topology regions using recombination breakpoint analysis (fig. 2), which also identified an intergenic region with standard topology within the *eba-181* anomalous topology gene cluster (fig. 2*a*).

**Fig. 2.**
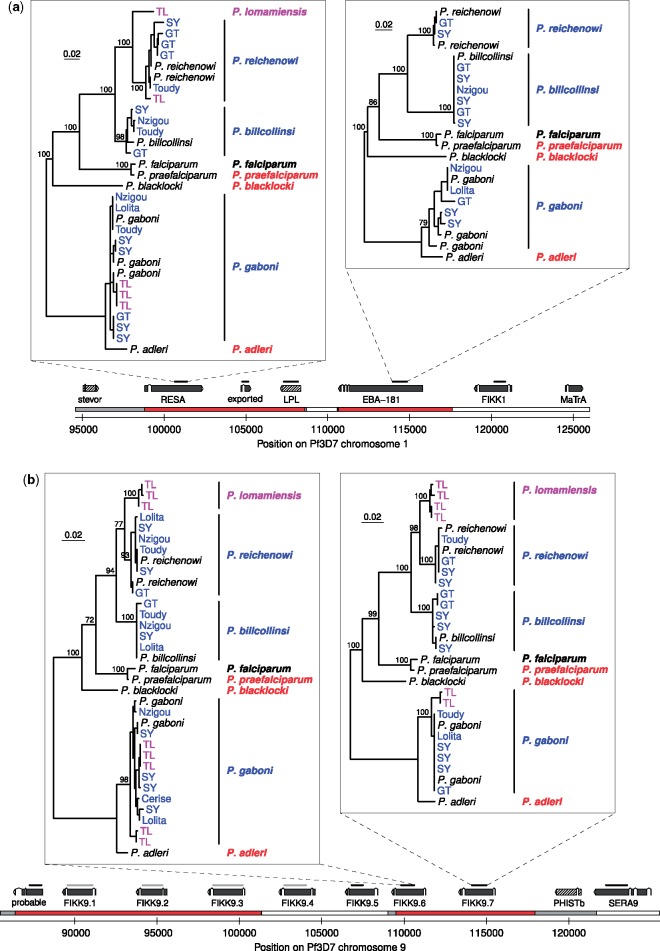
—Chromosomal regions revealing an ancient introgression event. (*a*, *b*) Regions on chromosomes 1 and 9, respectively. On the chromosome bar, regions with the standard topology are shown in white, regions with the anomalous topology in red; gray indicates topology is unclear. Above this, the positions of exons are shown for genes (solid gray) and pseudogenes (hatched), along with the name of the putative gene product. Bars above the genes indicate regions for which trees were generated, in black (PCR amplicons, newly assembled contigs, and published data) or gray (newly assembled contigs and published data only); four of these are shown in the upper panels, whereas additional trees are shown in supplementary Material online. Coloring and labeling of trees are as for fig. 1d. Scale bars represent 0.02 nucleotide substitutions per site; bootstrap values from 100 replicates are shown for interspecies nodes with at least 70% support; trees are rooted between the *P. gaboni* plus *P. adleri* clade and the other species. Exported, *Plasmodium* exported protein, unknown function; LPL, lysophospholipase; probable, probable protein, unknown function. On chromosome 9, *phistb* is absent from both the *P. reichenowi* and *P. billcollinsi* genome assemblies.

For these genes, no sequences among the PCR-derived amplicons or the newly generated contigs were consistent with the standard *Laverania* relationships for *P. reichenowi* and *P. billcollinsi*. Considered together with the relatively long branches separating the two species, these findings suggest that the event(s) giving rise to the anomalous topology occurred between the ancestors of *P. reichenowi* and *P. billcollinsi*, rather than between members of these two species in the recent past. This conclusion was strengthened by the analysis of six genes amplified from the bonobo parasite *Plasmodiumlomamiensis* (Liu et al. 2017), which showed the conventional close relationship of this species with *P. reichenowi* (fig. 2 and supplementary figs. 2*a*, 2*e*, and 3*a*, 3*f*–*h*, Supplementary Material online), indicating that gene transfer occurred before the divergence of their ancestors.

The finding of additional genes with the anomalous *P. billcollinsi–P. reichenowi* topology suggested that there might be still further genes with this topology. We thus used a less stringent method than Otto *et al.* to identify additional orthologues from elsewhere in the genome, reasoning that any of potential interest could be verified manually. We aligned 4,990 genes and scanned them for good support for a *P. billcollinsi–P. reichenowi* clade, followed by inspection of chromosomal alignments to confirm likely synteny (see supplementary methods, Supplementary Material online). This search identified 14 additional genes that may have undergone gene transfer between the ancestors of *P. billcollinsi* and *P. reichenowi*, in eight clusters on chromosomes 3, 4, 7, 9 and 12 (supplementary table 1, Supplementary Material online). These subtelomeric genes belong to multi-member gene families, group in small clusters, and in some cases are located on unplaced contigs in one or more species. Although evidence for the anomalous topology for each gene individually was weaker than for those in the *eba-181* and *fikk9.7* regions, it seems clear that genes with this evolutionary history are found at several loci outside the two main clusters.

Our new analyses indicate that the extent of gene transfer between *P. reichenowi* and *P. billcollinsi* is much greater than originally reported, with affected genes observed on six different chromosomes. This pattern could be the result of repeated horizontal gene transfer events, but it seems more plausible that all occurrences of this topology result from a single hybridization event followed by repeated backcrossing to one of the two parent lineages. Comparisons of genetic distances among *P. billcollinsi*, *P. reichenowi*, and *P. praefalciparum* suggest that introgression occurred from the common ancestor of *P. reichenowi* and *P. lomamiensis* to the *P. billcollinsi* lineage (supplementary note 2 and table 2, Supplementary Material online); this occurred before the divergence of *P. reichenowi* and *P. lomamiensis*, and therefore likely several million years ago (Liu et al. 2017). All the genes with the introgressed topology are located near chromosome ends, consistent with the idea that genes located there are more likely to lose linkage with loci encoding incompatibilities between species and are therefore more likely to be retained after hybridization (Barton and Bengtsson 1986).

Despite examination of nearly 5,000 genes from seven *Laverania* species, only one instance of horizontal gene transfer and one putative introgression event have been detected. This implies that successful hybridization is an extremely rare event. However, our findings also indicate that the barriers to gene flow between different parasite species are not absolute. Interestingly, most of the genes identified here as having introgressed (supplementary table 1, Supplementary Material online) appear to encode proteins that are exported and may be involved in erythrocyte remodeling (e.g., FIKK proteins and PHIST proteins) or in other interactions with host cells (erythrocyte-binding antigen 181, SURFIN4.2). Thus, gene transfer between *Plasmodium* species may have played a much greater role in the evolution of their host interactions than previously realized. It is therefore important to understand the circumstances under which *Plasmodium* species are able to hybridize, and whether such events can influence their pathogenicity, host tropism, and zoonotic potential.

## Materials and Methods

New *Laverania* sequences were obtained by amplification from ape blood and fecal samples using limiting dilution PCR (primer sequences in supplementary table 3, Supplementary Material online), and by extracting and reassembling sequencing reads obtained by Otto *et al.* that mapped to the genes of interest. Additional orthologue groups were added to those used by Otto et al. (2018) using OrthoMCL. Neighbor-joining trees for each group generated from uncorrected pairwise nucleotide distances were scanned for topologies in which *P. reichenowi* and *P. billcollinsi* clustered together, and were considered further if a maximum likelihood tree indicated at least 95% support for the relevant clade in either nucleotide or protein trees, and if inspection of chromosome alignments confirmed likely orthology. Maximum likelihood trees were obtained using RAxML v8.2.12. Recombination breakpoints were detected in alignments of chromosome regions using DualBrothers implemented in Geneious (for more details, see Supplementary Material online).

## Supplementary Material

Supplementary data are available at *Genome Biology and Evolution* online.

## Supplementary Material

evz244_Supplementary_Data

## Notes

**Data deposition:** This project has been deposited at NCBI GenBank under the accession numbers MK491955–MK492101 and MK510915. Details are given in supplementary table S4, Supplementary Material online.
