# Supplementary material for: Ancient Introgression between Two Ape Malaria Parasite Species
Source: Genome Biol Evol. 2019 Nov 7;11(11):3269–74. doi: 10.1093/gbe/evz244 (PMC7145702; doi:10.1093/gbe/evz244)
Supplement: evz244_Supplementary_Data [file evz244_supplementary_data.zip › GBE Supplement.docx]

**Supplementary Materials**

**Ancient introgression between two ape malaria parasite species**

Lindsey J. Plenderleith^1^, Weimin Liu^2^, Gerald H. Learn^2^, Dorothy E. Loy^2,3^, Sheri Speede^4^, Crickette M. Sanz^5,6^, David B. Morgan^6,7^, Paco Bertolani^8^, John A. Hart^9^, Terese B. Hart^9^, Beatrice H. Hahn^2,3^ and Paul M. Sharp^1^*

^1^Institute of Evolutionary Biology, and Centre for Immunity, Infection and Evolution, University of Edinburgh, United Kingdom

^2^Department of Medicine, University of Pennsylvania, Philadelphia

^3^Department of Microbiology, University of Pennsylvania, Philadelphia

^4^Sanaga-Yong Chimpanzee Rescue Center, International Development Association-Africa, Portland, Oregon

^5^Department of Anthropology, Washington University in St. Louis, St Louis, Missouri

^6^Wildlife Conservation Society, Congo Program, Brazzaville, Republic of the Congo

^7^Lester E. Fisher Center for the Study and Conservation of Apes, Lincoln Park Zoo, Chicago, Illinois

^8^Leverhulme Centre for Human Evolutionary Studies, University of Cambridge, United Kingdom

^9^Lukuru Wildlife Research Foundation, Tshuapa-Lomami-Lualaba Project, Kinshasa, Democratic Republic of the Congo

*Corresponding author: E-mail: [paul.sharp@ed.ac.uk](mailto:paul.sharp@ed.ac.uk)

**
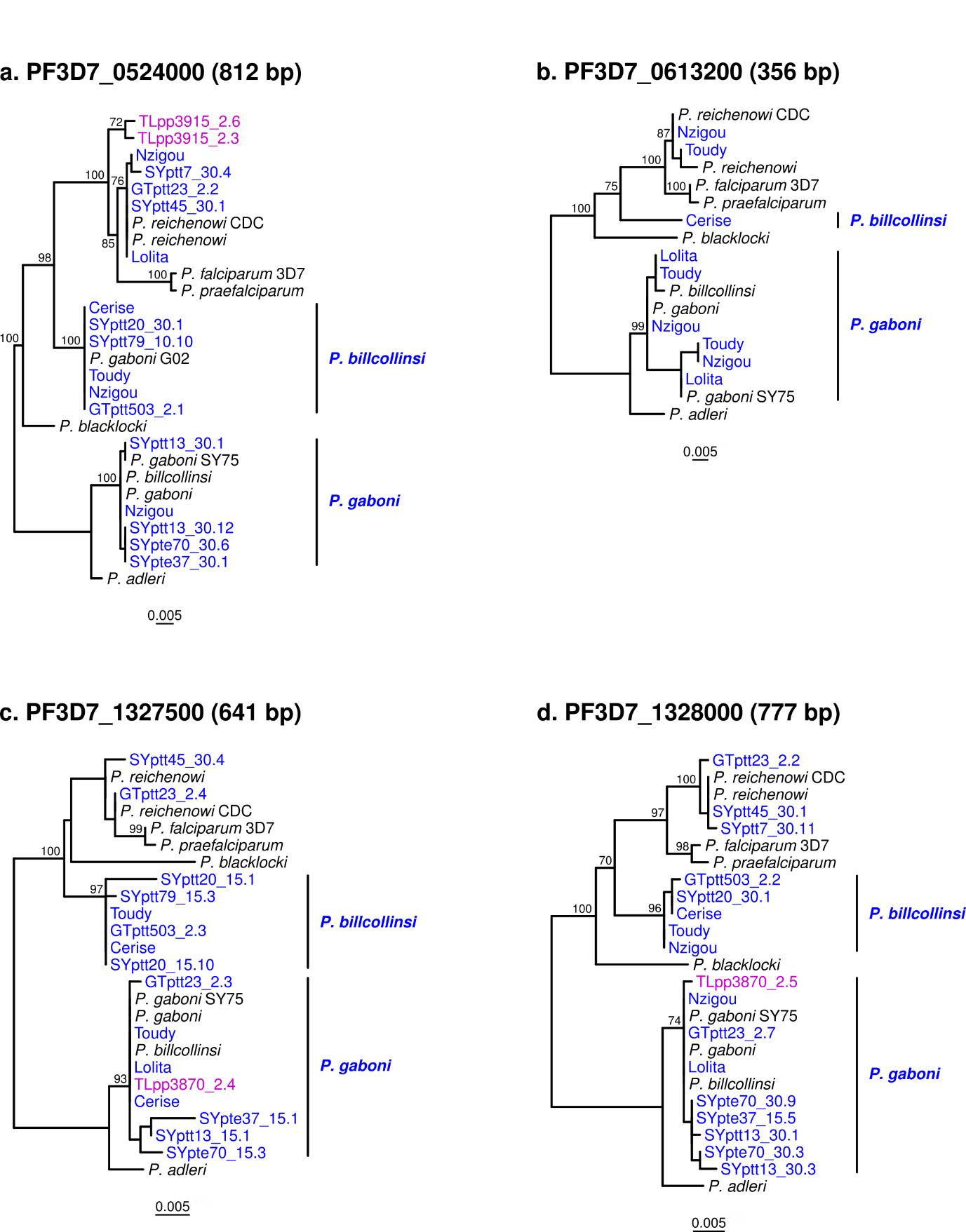
**

**Supplementary Fig. S1.** Genes previously hypothesised to have transferred from *P. gaboni* to *P. billcollinsi* (“topology C” in Otto et al. 2018). Phylogenetic trees were constructed using maximum likelihood methods, for orthologues of **a**, PF3D7_0524000 (karyopherin beta), **b**, PF3D7_0613200 (conserved *Plasmodium* protein, unknown function), **c,** PF3D7_1327500 (conserved protein, unknown function), **d**, PF3D7_1328000 (conserved *Plasmodium* protein, unknown function). The tree in panel c is reproduced from Fig. 1d to show details of sequence names. In all four trees, the *P. billcollinsi* reference sequence clusters with *P. gaboni*, but all other likely *P. billcollinsi* sequences fall in a position consistent with the standard species tree (Fig. 1a). The trees in panels a and c display poor resolution of the *P. falciparum* sub-clade, owing to the small number of informative sites in the alignments, apparent in the short branch lengths, but this does not affect the conclusion relating to *P. billcollinsi*. Sequences were obtained from the published genome assemblies (black labels, reference strains from Otto et al. unless indicated otherwise), from new contigs generated by reassembly of sequencing data released by Otto et al. (labelled with the name of the animal from which the data were derived: Toudy, Nzigou, Cerise, Lolita; see Supplementary Table 1 of Otto et al. 2018), or from limiting dilution PCR of ape blood and faecal samples. PCR-derived sequences are labelled with a two-letter code denoting the field site of origin (SY, Sanaga-Yong Chimpanzee Rescue Center; GT, Goualougo Triangle; TL, Tshuapa–Lomami–Lualaba), lower case letters indicating the host species origin (ptt, *Pan troglodytes;* pte, *P. t. ellioti*; pp, *Pan paniscus*), the sample number, PCR dilution and well position. For PCR-derived sequences and new contigs, colour indicates whether the sample was obtained from chimpanzee (blue) or bonobo (purple). Sequences presumed to originate from *P. billcollinsi* and *P. gaboni* are indicated by brackets at the right. Note that in addition to the anomalous position of the *P. billcollinsi* reference genome sequences, for one gene (part a) there is a sequence from the *P. gaboni* G02 genome assembl (Otto et al. 2018) that is identical to true *P. billcollinsi* sequences, presumably reflecting another assembly artefact. Scale bars represent 0.005 nucleotide substitutions per site; bootstrap values from 100 replicates are shown for interspecies nodes with at least 70% support; trees are rooted between the *P. gaboni* plus *P. adleri* clade and the other species; numbers in brackets indicate the number of nucleotides in the alignment in *P. falciparum*.


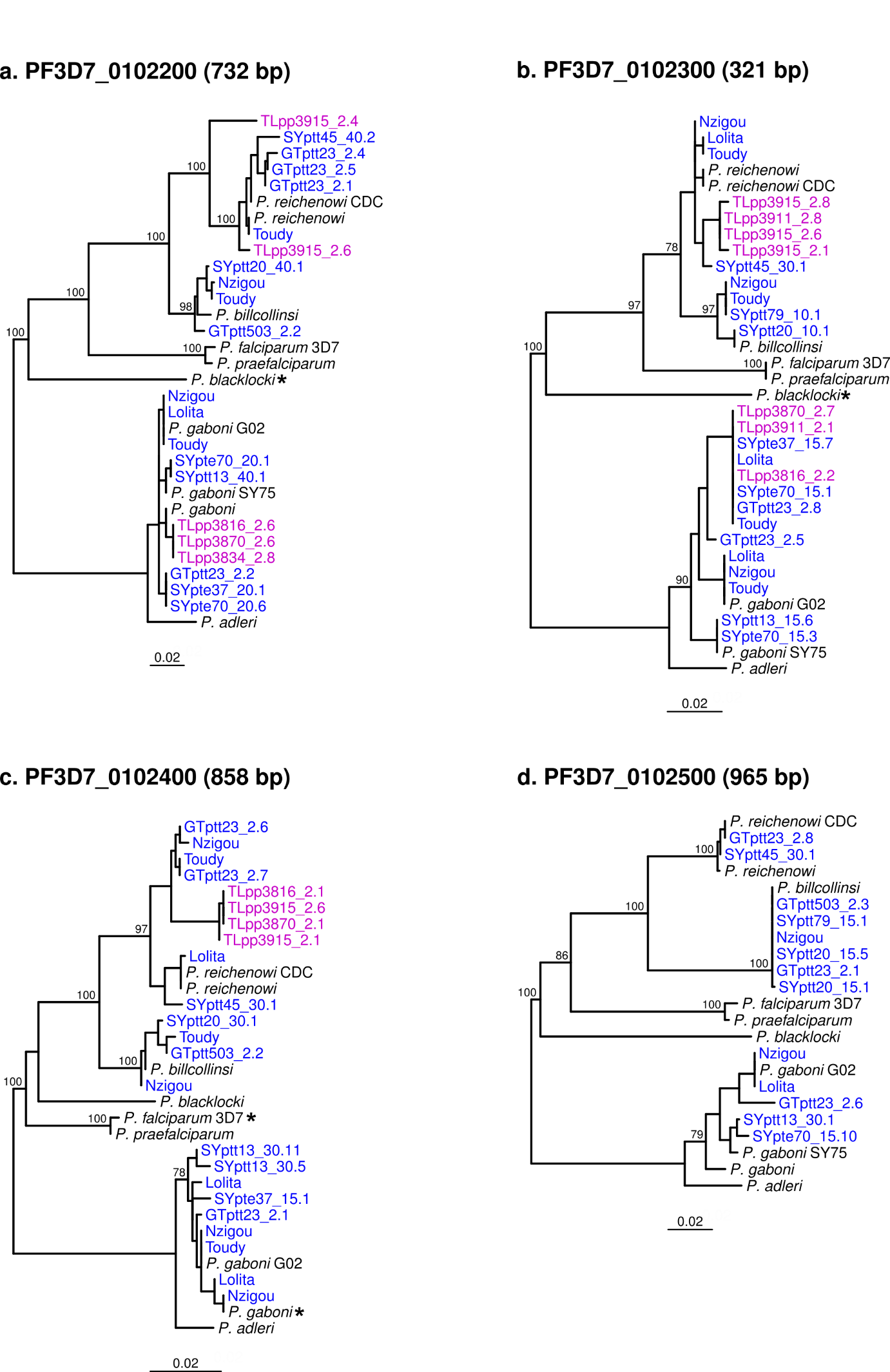


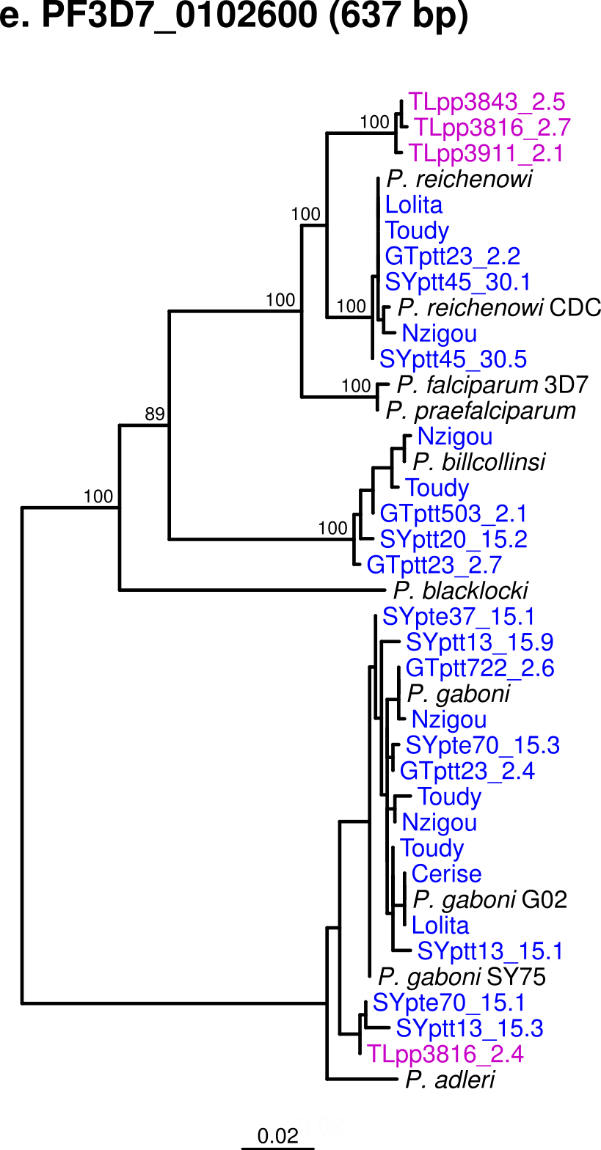


**Supplementary Fig. S2.** Phylogenetic trees for genes from the *eba-181* region on chromosome 1. (This region is illustrated in Fig. 2a.) Phylogenetic trees were constructed using maximum likelihood methods, for orthologues of **a,** PF3D7_0102200 (ring-infected erythrocyte surface antigen, RESA), **b**, PF3D7_0102300 (*Plasmodium* exported protein, unknown function), **c,** PF3D7_0102400 (lysophospholipase, putative), **d**, PF3D7_0102500 (erythrocyte binding antigen-181, EBA-181), **e**, PF3D7_0102600 (FIKK1). Trees in panels a-d exhibit the anomalous topology with *P. reichenowi* and *P. billcollinsi* clustered (as portrayed in Fig. 1c); the tree in panel e exhibits the standard species topology (as in Fig. 1a). Trees in panels a and d are reproduced from Fig. 2a to show details of sequence names. Colouring and labelling of sequences are as described for Supplementary Fig. 1. An asterisk beside the reference sequence indicates the orthologue is a pseudogene in at least some sequences from that species. Scale bars represent 0.02 nucleotide substitutions per site; bootstrap values from 100 replicates are shown for interspecies nodes with at least 70% support; trees are rooted between the *P. gaboni* plus *P. adleri* clade and the other species; numbers in brackets indicate the number of nucleotides in the alignment in *P. falciparum*.


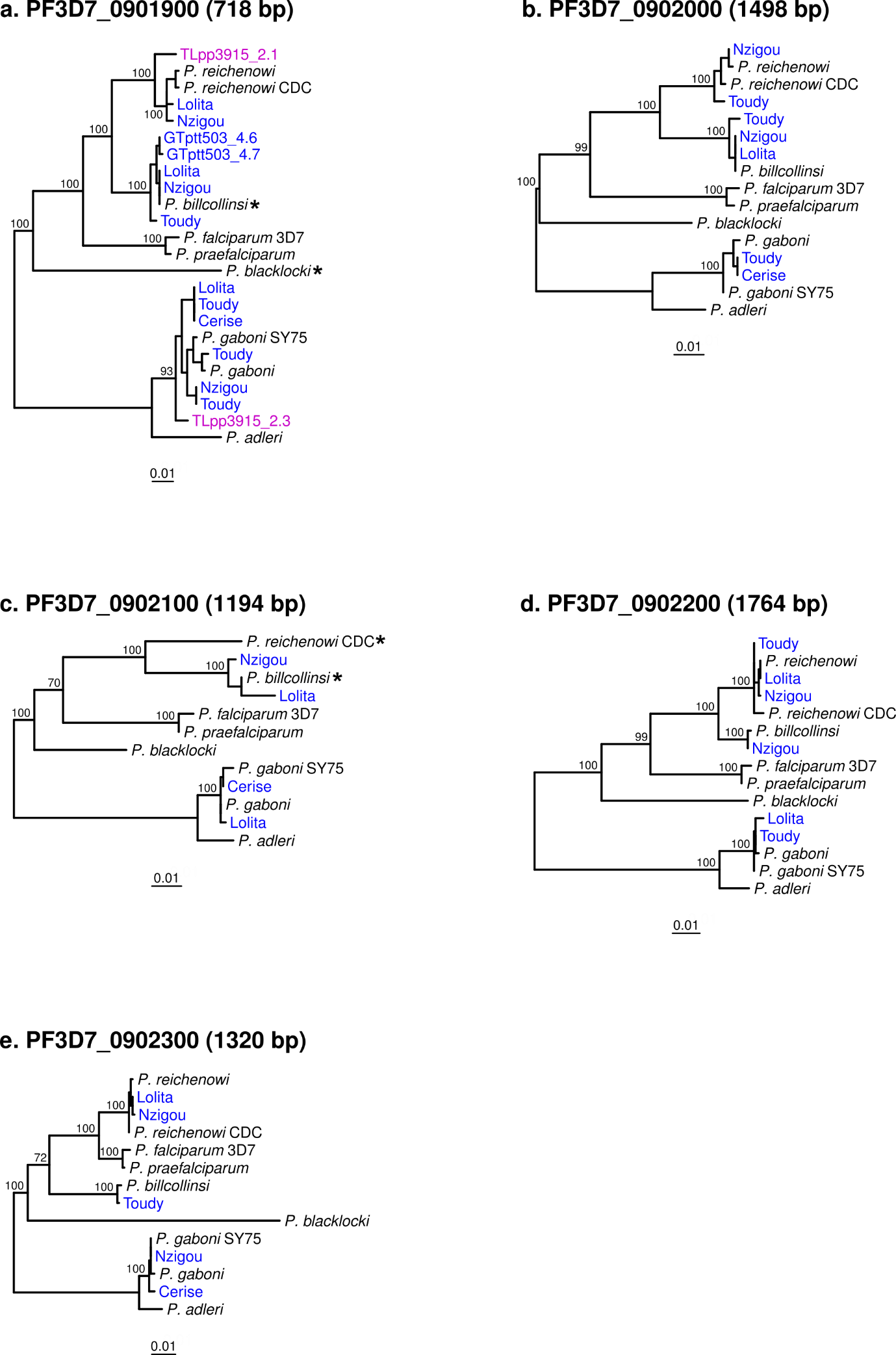


**
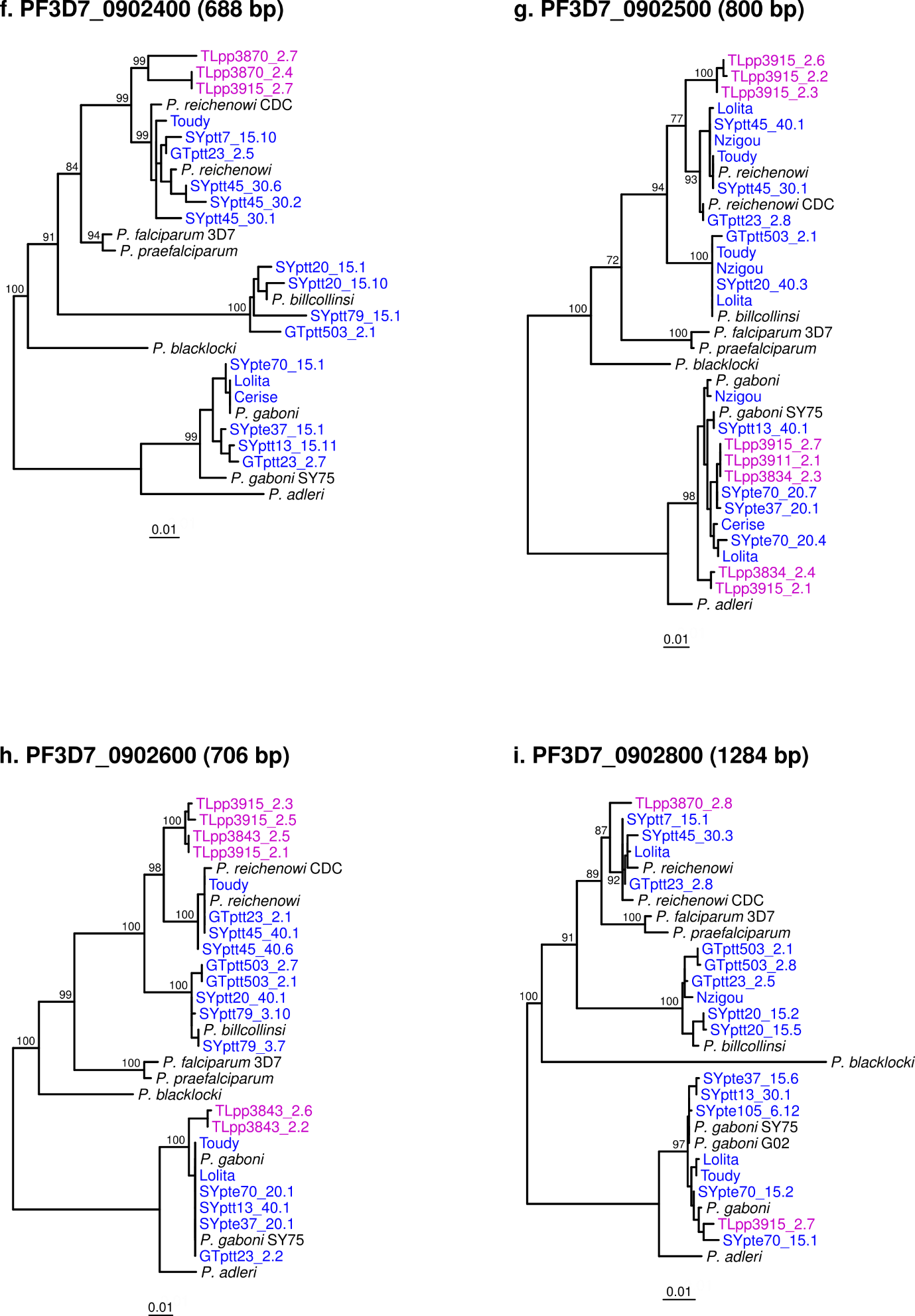
**

**Supplementary Fig. S3.** Phylogenetic trees for genes from the *fikk9.7* region on chromosome 9. (This region is illustrated in Fig. 2b.) Phylogenetic trees were constructed using maximum likelihood methods, for orthologues of **a,** PF3D7_0901900 (probable protein, unknown function), **b,** PF3D7_0902000 (FIKK9.1), **c,** PF3D7_0902100 (FIKK9.2), **d**, PF3D7_0902200 (FIKK9.3), **e**, PF3D7_0902300 (FIKK9.4), **f**, PF3D7_0902400 (FIKK9.5), **g**, PF3D7_0902500 (FIKK9.6), **h**, PF3D7_0902600 (FIKK9.7), **i**, PF3D7_0902800 (serine repeat antigen 9, SERA9). Trees in panels a-d, g and h exhibit the anomalous topology with *P. reichenowi* and *P. billcollinsi* clustered (as depicted in Fig. 1c); the trees in panels e, f and i exhibit the standard species topology (as in fig. 1a). PF3D7_0902700 (PHISTb, pseudogene) is not shown because it is absent from the *P. reichenowi* and *P. billcollinsi* assemblies. Trees in panels g and h are reproduced from fig. 2b to show details of sequence names. An asterisk beside the reference sequence indicates the orthologue is a pseudogene in at least some sequences from that species. Scale bars represent 0.02 nucleotide substitutions per site; bootstrap values from 100 replicates are shown for interspecies nodes with at least 70% support; trees are rooted between the *P. gaboni* plus *P. adleri* clade and the other species; numbers in brackets indicate the number of nucleotides in the alignment in *P. falciparum*.

**
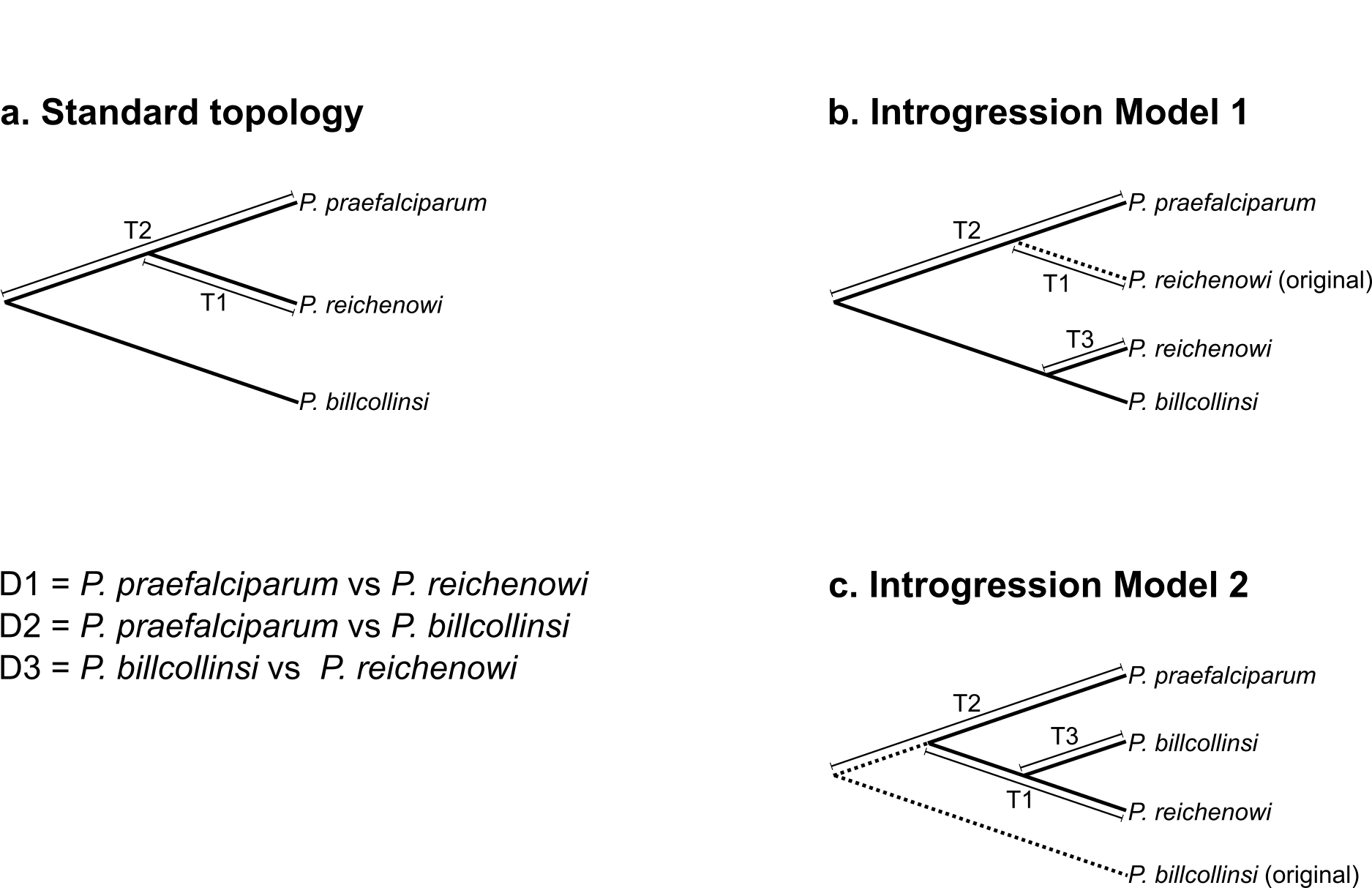
**

**Supplementary Fig. S4.** Relationships among *P. praefalciparum*, *P. reichenowi* and *P. billcollinsi*. **a**, Relationships in the standard species topology. T1 represents the time since the common ancestor of *P. praefalciparum* and *P. reichenowi*; T2 represents the time since the common ancestor of *P. praefalciparum* and *P. billcollinsi*. **b**, Relationships under a model of introgression from *P. billcollinsi* into *P. reichenowi* (model 1). **c**, Relationships under a model of introgression from *P. reichenowi* into *P. billcollinsi* (model 2). In parts b and c, T3 represents the time since the hybridisation between *P. reichenowi* and *P. billcollinsi*. Dotted lines indicate distances that are no longer obtainable from the data. Distances are not to scale.


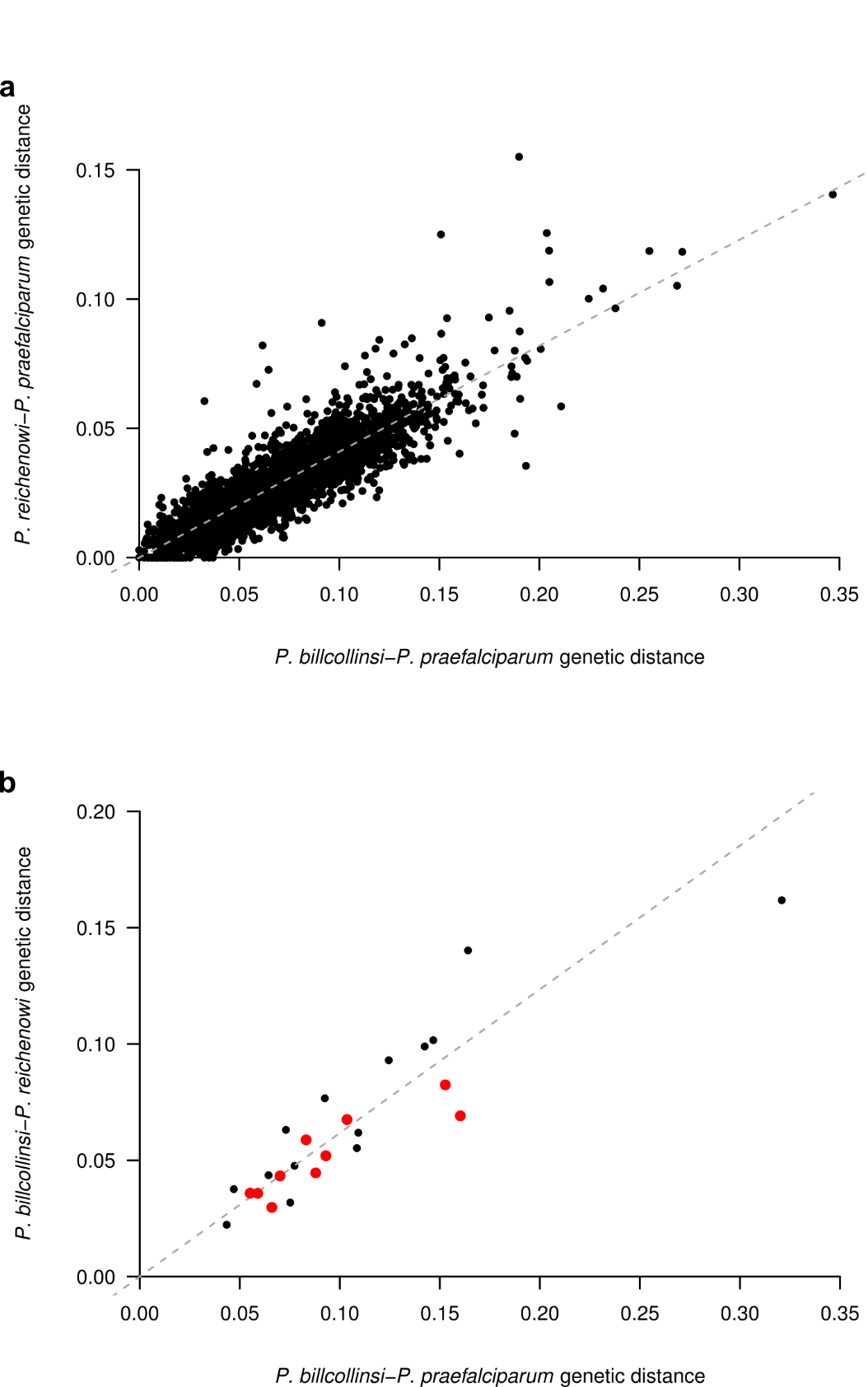


**Supplementary Fig. S5.** Pairwise genetic distances among *P. praefalciparum*, *P. reichenowi* and *P. billcollinsi*. **a**, Genes with the standard topology. For each gene, the distance between *P. reichenowi* and *P. praefalciparum* (D1) is plotted against the distance between *P. billcollinsi* and *P. praefalciparum* (D2). The dotted line through the origin has slope equal to the weighted mean (by alignment length) of D1 divided by the weighted mean of D2; the strong correlation between D1 and D2 justifies the assumption of a molecular clock and the equivalence of D1/D2 with T1/T2 (the ratio of times since the common ancestors). **b**, Genes with the anomalous *P. reichenowi*-*P. billcollinsi* topology. For each gene, the distance between *P. reichenowi* and *P. billcollinsi* (D3) is plotted against the distance between *P. praefalciparum* and *P. billcollinsi* (D2). The dotted line through the origin has slope equal to the weighted mean of D3 divided by the weighted mean of D2; again, the strong correlation of D2 and D3 justifies the assumption of a molecular clock. Red points represent genes from the *eba-181* and *fikk9.7* clusters; black points represent genes from other locations.

**Supplementary Methods**

Ape samples

Small amounts of blood or dried blood spots were obtained from sanctuary chimpanzees cared for at the Sanaga-Yong (SY) Chimpanzee Rescue Center in Cameroon, all of which were collected for veterinary purposes or represented leftover specimens from yearly health examinations (Sundararaman et al. 2016). Faecal samples from wild-living chimpanzees and bonobos were collected at field sites in the Republic of Congo (GT) and the Democratic Republic of the Congo (TL), respectively (Liu et al. 2010; 2017). All samples were collected with the approval of the respective government agencies and shipped in compliance with Convention on International Trade in Endangered Species of Wild Fauna and Flora regulations and country-specific import and export permits as previously described (Sundararaman et al. 2016; Liu et al. 2010; 2017).

Single template amplification of ape parasites

*Laverania* sequences were amplified using limiting dilution PCR from ape blood and faecal samples previously identified as containing these parasites (Liu et al. 2016), using newly designed primer sequences (see supplementary table S3 for primer sequences).

Assembly of new contigs

For each gene, read files from samples reported by Otto et al. (2018) to consist of at least 2% *P. billcollinsi* sequences^1^ (Cerise, Lolita, Toudy and Nzigou; see Supplementary Table 1 of Otto et al. 2018) were mapped to the orthologue and flanking sequence from the *P. falciparum* reference genome (Pf3D7v3) using smalt (https://www.sanger.ac.uk/science/tools/smalt-0; minimum identity parameter 0.8 or 0.9, with the value giving the best results for each read set being used in each case). Mapped reads were extracted and assembled *de novo* into contigs using the assembler in Geneious v11.1.4 with default “medium sensitivity/fast” settings. Highest quality contigs for each sample, generally greater than 900 bp in length with average read coverage >11, were aligned using MAFFT v7 (Katoh and Standley 2013) as implemented in Geneious. Longest contigs that aligned most frequently (visually assessed) were selected and aligned with MAFFT to *Laverania* reference genomes to identify exon locations. Contigs from the reference genomes that were identical to published assemblies were not included.

Phylogenetic analyses

Sequences from published genomes (supplementary table S5), from newly-assembled contigs, and from limiting dilution derived PCR amplicons were aligned with MAFFT, manually inspected and corrected, and used to generate maximum likelihood trees using RaxML v8.2.12 (Stamatakis 2014) with 100 bootstrap replicates (model GTRGAMMA). Phylogenetic trees were rooted between the *P. gaboni* plus *P. adleri* clade and the other *Laverania* species, and visualised using the ape (Paradis et al. 2004) and phangorn R packages (Schliep 2011).

Recombination breakpoint analysis

Syntenic chromosome regions, beginning with the telomere-proximal intergenic sequence of the first anomalous gene and ending with the first standard topology gene, were extracted from published assemblies of *P. falciparum*, *P. reichenowi*, *P. billcollinsi* and either *P. gaboni* (*eba-181* region) or *P. blacklocki* (*fikk9.7* region), depending on availability, and aligned with MAFFT. Large regions with gaps in two or more species were excluded from the alignment. Recombination breakpoints in the four-species alignments were detected using DualBrothers (Minin et al. 2005) as implemented in Geneious Prime v2019.0.4, using a scanning window length of 500 bp and otherwise default options.

Identification of additional genes with anomalous topology

Orthologue groups were based on those in the “Lav7sp” orthologue table from Otto et al. (2018). To obtain additional orthologue groups, gene models from the new *Laverania* genomes and *P. falciparum* strain 3D7 were translated and used in an all-against-all blastp search with masking of low complexity sequence and an e-value cutoff of 10^-5^. Gene groups were produced using OrthoMCL v2.0.9 (Li et al. 2003), and retained as orthologue groups if no species appeared more than once in the group. Further orthologue groups were obtained from groups with apparent paralogues, if these groups could be resolved based on chromosome placement or by excluding paralogues on unplaced contigs. Nucleotide sequences for each orthologue group were aligned using Prank v.170427 (Loytynoja 2014) with the -F flag, then sequences of less than 50% of the median length were excluded and remaining positions with alignment or assembly gaps were removed. The ape package in R^8^ was used to calculate pairwise, uncorrected genetic distances between species, calculate neighbour-joining trees, and scan the trees for topologies in which *P. reichenowi* and *P. billcollinsi* clustered together. Unresolved paralogue groups were examined by alignment of the entire group followed by manual inspection of a RAxML maximum likelihood tree to identify clustering of *P. billcollinsi* and *P. reichenowi* within a sub-clade of likely syntenic orthologues, ignoring additional copies on unplaced contigs. Orthologue groups without *P. falciparum* sequences were examined for anomalous topology but only considered further if they formed a cluster on the chromosome with another anomalous topology gene that did have a *P. falciparum* orthologue. For candidate anomalous topology genes, maximum likelihood nucleotide trees were generated from alignments using RAxML (model GTRGAMMA) and bootstrap values calculated from 100 replicates. Maximum likelihood protein trees were obtained by translating coding sequences, aligning with MUSCLE (Edgar 2004), and inferring trees with RAxML (model PROTGAMMAJTTF, bootstrap values from 100 replicates). Genes were excluded from the analysis if the *P. billcollinsi-P. reichenowi* clade did not have at least 95% bootstrap support in either the nucleotide or protein tree, unless adjacent genes also had this topology with good support. Alignments of concatenated assemblies of *P. falciparum*, *P. reichenowi* and *P. billcollinsi* were then inspected to confirm that putative orthologues were likely to be syntenic. A gene was retained if it was located in the same chromosome position as the putative orthologue in *P. falciparum*, or located on an unplaced contig with at least one neighbouring gene with a match to the neighbouring gene in *P. falciparum*. Orthologue groups with no *P. falciparum* sequences were inspected in *P. praefalciparum* instead. If putative orthologues had good blastn matches elsewhere in the genome (covering at least 75% of the gene, and no more than 5% lower identity than the match to the putative orthologue), the additional genes were incorporated into the trees, and the region was excluded if this affected the conclusion of gene transfer. Genes with the anomalous topology of the previously reported *rh5* cluster on chromosome 4 (Sundararaman et al. 2016) were searched for in the same way, except that the trees were scanned for topologies in which *P. falciparum*, *P. praefalciparum*, *P. adleri* and *P. gaboni* were monophyletic; however, no new genes with this topology were identified.

Genetic distance analysis

Coding sequences for all genes with the *P. billcollinsi-P. reichenowi* anomalous topology (supplementary table S1) were realigned using TranslatorX (Abascal et al. 2010) followed by manual correction. For standard topology genes, genetic distances were obtained by extracting *P. billcollinsi*, *P. reichenowi* and *P. praefalciparum* from existing Prank alignments of orthologue groups and masking ambiguous sites, excluding genes with anomalous topologies and outlier genes found to have problematic alignments. Corrected pairwise distances were calculated using the ape package in R (TN93+gamma model).

**Supplementary Note 1**

Molecular epidemiological studies of wild chimpanzee (*Pan troglodytes*), bonobo (*Pan paniscus)*, and western (*Gorilla gorilla*) and eastern (*Gorilla beringei*) gorilla populations have shown that they harbour a plethora of *Plasmodium* parasites, the most common of which are members of the *Laverania* subgenus (Ollomo et al. 2009; Liu et al. 2010, 2017; Boundenga et al. 2015). There are seven *Laverania* species in nonhuman great apes, termed *P. lomamiensis, P. reichenowi*, *P. praefalciparum*, *P. billcollinsi*, *P. blacklocki*, *P. gaboni* and *P. adleri* (fig. 1a), which exhibit different host tropisms (Liu et al. 2010, 2017; Prugnolle et al. 2011; Boundenga et al. 2015). *P. lomamiensis* seems to infect only bonobos (purple in fig. 1a), and only in a small part of their natural range east of the Lomami River (Liu et al. 2017). *P. reichenowi*, *P. billcollinsi* and *P. gaboni* are considered chimpanzee parasites (blue in fig. 1a), because they are highly prevalent in all four chimpanzee subspecies, but are absent from sympatric gorillas. However, *P. gaboni* and *P. reichenowi* have also been found in wild bonobos (figs. 1d and 2; supplementary figs. S1-S3), indicating occasional cross-species transmissions of these parasites from neighbouring chimpanzee communities (Liu et al. 2017). *P. praefalciparum*, *P. adleri* and *P. blacklocki* are highly prevalent in western gorillas, but do not infect sympatric chimpanzees. They are thus considered gorilla parasites (red in fig. 1a), although they have not been found in eastern gorillas, which seem to be free of *Laverania* infections (Liu et al. 2010).

**Supplementary Note 2**

A schematic representation of the species history of *P. praefalciparum*, *P. reichenowi* and *P. billcollinsi* is shown in supplementary fig. S4a; the time since the common ancestor of *P. praefalciparum* and *P. reichenowi* is denoted by T1, and time since the common ancestor of all three species by T2. The anomalous topology, in which *P. billcollinsi* and *P. reichenowi* are each other’s closest relative, implies one of two events: introgression from *P. billcollinsi* into *P. reichenowi* (supplementary fig. S4b, model 1), or introgression from *P. reichenowi* into *P. billcollinsi* (supplementary fig. S4c, model 2). In either scenario, there was a hybridisation event, at time T3, which must have occurred after the divergence of *P. praefalciparum* and *P. reichenowi*. Therefore T3 must be less than T1.

To examine these relationships in the observed data, pairwise interspecies genetic distances were calculated between *P. praefalciparum* and *P. reichenowi* (D1), *P. praefalciparum* and *P. billcollinsi* (D2) and *P. billcollinsi* and *P. reichenowi* (D3). For genes with the standard topology, D1 and D2 varied considerably among genes, but there was a strong correlation between the two pairwise distances (supplementary fig. S5a). The average ratio of D1/D2 was calculated as the mean value of D1 divided by the mean value of D2, each weighted by alignment length (supplementary table S2; supplementary fig. S5a). Similarly, for the genes with the anomalous topology (supplementary table S6), there was a good correlation between D2 and D3 (supplementary table S2; supplementary fig. S5b); the average value of D3/D2 = 0.62.

Under the assumption of a molecular clock, justified by the strong correlation between D1 and D2, the value of D1/D2 (= 0.41) would be equivalent to the T1/T2 ratio. For the introgressed genes, under model 1 (supplementary fig. S4b), the D3/D2 ratio (= 0.62) would be equivalent to T3/T2. However, since T2 is the denominator in both ratios, these values are not consistent with the expectation that T3 < T1. That is, for the introgressed genes, the value of D3 appears to be too high, relative to the value of D2, to reflect transfer from *P. billcollinsi* to *P. reichenowi*.

For the introgressed genes, under model 2 (supplementary fig. S4c), the D3/D2 ratio would be equivalent to T3/T1. Given that T1/T2 has been estimated to be 0.41, this implies that here T3/T2 would be 0.62 x 0.41, or ~0.25. This value is consistent with the expectation that T3 < T1. Thus we conclude that model 2, with introgression from *P. reichenowi* to *P. billcollinsi*, is the more likely scenario.

If only the genes from the better-supported *eba-181* and *fikk9.7* clusters (red in supplementary fig. S5b) were used to calculate D3/D2 (supplementary table S2), this ratio becomes 0.52, and the conclusion is unchanged. The implication of these D3/D2 ratios is that the introgression event occurred at a time about halfway back to the common ancestor of *P. praefalciparum* and *P. reichenowi*.

**Literature Cited**

Abascal F, Zardoya R, Telford MJ. 2010. TranslatorX: multiple alignment of nucleotide sequences guided by the amino acid translations. Nucleic Acids Res. 38:W7-W13.

Boundenga L, et al. 2015. Diversity of malaria parasites in great apes in Gabon. Malaria J. 12:111.

Edgar RC. 2004. MUSCLE: multiple sequence alignment with high throughput and accuracy. Nucleics Acids Res. 32:1792-1797.

Katoh K, Standley DM. 2013. MAFFT multiple sequence alignment software version 7: improvements in performance and usability. Mol Biol Evol. 30:772-780.

Li L, Stoeckert CJ, Roos DS. 2003. OrthMCL: identification of ortholog groups for eukaryotic genomes. Genome Res. 13:2178-2189.

Liu W, et al. 2010. Origin of the human malaria parasite *Plasmodium falciparum* in gorillas. Nature 467:420-425.

Liu W, et al. 2016. Multigenomic delineation of *Plasmodium* species of the *Laverania* subgenus infecting wild-living chimpanzees and gorillas. Genome Biol Evol. 6:1929-1939.

Liu W, et al. 2017. Wild bonobos host geographically restricted malaria parasite including a putative new *Laverania* species. Nat Commun. 8:1635.

Loytynoja A. 2014. Phylogeny-aware alignment with PRANK. Methods Molec Biol. 1079:155-170.

Ollomo B, et al. 2009. A new malaria agent in African hominids. PLoS Pathog. 5:e1000446.

Otto TD, et al. 2018. Genomes of all known members of a *Plasmodium* subgenus reveal paths to virulent human malaria. Nat Microbiol. 3:687-697.

Paradis E, Claude J, Strimmer K. 2004. APE: Analyses of Phylogenetics and Evolution in R language. Bioinformatics 20:289-290.

Prugnolle F, et al. 2011. A fresh look at the origin of *Plasmodium falciparum*, the most malignant malaria agent. PLoS Pathog. 7:e1001283.

Schliep KP. 2011. phangorn: phylogenetic analysis in R. Bioinformatics 27:592-593.

Stamatakis A. 2014. RAxML version 8: a tool for phylogenetic analysis and post-analysis of large phylogenies. Bioinformatics 30:1312-1313.

Sundararaman SA, et al. 2016. Genomes of cryptic chimpanzee *Plasmodium* species reveal key evolutionary events leading to human malaria. Nat Commun. 7:11078.
